# Chronic kidney disease in the type 2 diabetic patients: prevalence and associated variables in a random sample of 2642 patients of a Mediterranean area

**DOI:** 10.1186/1471-2369-13-87

**Published:** 2012-08-20

**Authors:** Gabriel Coll-de-Tuero, Manel Mata-Cases, Antonio Rodriguez-Poncelas, Josep MA Pepió, Pilar Roura, Belen Benito, Josep Franch-Nadal, Marc Saez

**Affiliations:** 1 Primary Healthcare Center Anglès, Department of Medical Sciences, University of Girona, Girona, Spain; 2 Primary Healthcare Center La Mina, Institut Catalá de la Salut, Barcelona, Spain; 3 Primary Healthcare Center Anglès, Research Unit, I.A.S, Salt, Spain; 4 Primary Healthcare Center Tortosa Oest, Tortosa, Spain; 5 Departement of Medical Sciences, University Autònoma of Barcelona, Barcelona, Spain; 6 Primary Healthcare Center, Raval Sud, Institut Catalá de la Salut, Barcelona, Spain; 7 Primary Healthcare Center, Raval Sud, Barcelona, Spain; 8 Applied Economics and Health(GRECS), Department of Economics, University of Girona, Girona, Spain

**Keywords:** Kidney disease, Renal impairment, Albuminuria, Diabetic nephropathy

## Abstract

**Background:**

Kidney disease is associated with an increased total mortality and cardiovascular morbimortality in the general population and in patients with Type 2 diabetes. The aim of this study is to determine the prevalence of kidney disease and different types of renal disease in patients with type 2 diabetes (T2DM).

**Methods:**

Cross-sectional study in a random sample of 2,642 T2DM patients cared for in primary care during 2007. Studied variables: demographic and clinical characteristics, pharmacological treatments and T2DM complications (diabetic foot, retinopathy, coronary heart disease and stroke). Variables of renal function were defined as follows: 1) Microalbuminuria: albumin excretion rate & 30 mg/g or 3.5 mg/mmol, 2) Macroalbuminuria: albumin excretion rate & 300 mg/g or 35 mg/mmol, 3) Kidney disease (KD): glomerular filtration rate according to Modification of Diet in Renal Disease < 60 ml/min/1.73 m^2^ and/or the presence of albuminuria, 4) Renal impairment (RI): glomerular filtration rate < 60 ml/min/1.73 m^2^, 5) Nonalbuminuric RI: glomerular filtration rate < 60 ml/min/1.73 m^2^ without albuminuria and, 5) Diabetic nephropathy (DN): macroalbuminuria or microalbuminuria plus diabetic retinopathy.

**Results:**

The prevalence of different types of renal disease in patients was: 34.1% KD, 22.9% RI, 19.5% albuminuria and 16.4% diabetic nephropathy (DN). The prevalence of albuminuria without RI (13.5%) and nonalbuminuric RI (14.7%) was similar. After adjusting per age, BMI, cholesterol, blood pressure and macrovascular disease, RI was significantly associated with the female gender (OR 2.20; CI 95% 1.86–2.59), microvascular disease (OR 2.14; CI 95% 1.8–2.54) and insulin treatment (OR 1.82; CI 95% 1.39–2.38), and inversely associated with HbA1c (OR 0.85 for every 1% increase; CI 95% 0.80–0.91). Albuminuria without RI was inversely associated with the female gender (OR 0.27; CI 95% 0.21–0.35), duration of diabetes (OR 0.94 per year; CI 95% 0.91–0.97) and directly associated with HbA1c (OR 1.19 for every 1% increase; CI 95% 1.09–1.3).

**Conclusions:**

One-third of the sample population in this study has KD. The presence or absence of albuminuria identifies two subgroups with different characteristics related to gender, the duration of diabetes and metabolic status of the patient. It is important to determine both albuminuria and GFR estimation to diagnose KD.

## Background

The concept of kidney disease is based on the presence of albuminuria and/or impaired renal function that lasts for more than three months [1]. Kidney disease is associated with an increased total mortality and cardiovascular morbimortality in the general population [2] and in patients with Type 2 diabetes (T2DM) [3]. Patients with T2DM with renal impairment have an increased mortality risk, specially a higher risk of cardiovascular (CV) death, when compared to other diabetic patients without renal impairment [4]. Moreover, albuminuria predicts an increased risk of myocardial infarction, stroke, CV death, total death and heart failure in patients with T2DM in comparison with non-diabetic patients [5]. However, not all T2DM patients with renal impairment have albuminuria [6]. Therefore, the National Kidney Foundation (NKF) [1] recommends the determination of plasma creatinine to estimate glomerular filtration rate (GFR), because renal impairment and albuminuria are independently associated with strokes [7]. The simultaneous presence of renal impairment and albuminuria is associated with an increased risk of CV morbidity, increased risk of total mortality, and progression of renal disease [8].

During a 15-year follow-up, the United Kingdom Prospective Diabetes (UKPDS) study showed that 5% of the patients with T2DM had a 2-fold increase in creatinine levels, 20% had macroalbuminuria defined as a urinary albumin concentration ≥ 300 mg/l, 40% developed renal impairment, 45% developed microalbuminuria defined as a urinary albumin concentration 50–299 mg/l and 75% experienced a CV event [9]. Early detection of kidney disease in patients with diabetes is important because close monitoring of cardiovascular risk factors, and specific drugs acting on the renin-angiotensin system slow down the progression of renal disease [10].

There have been several reports on the prevalence of kidney disease and forms of renal disease in patients with T2DM of Mediterranean countries [11-13]. Only one study has been conducted in a randomly selected population sample in Spain [14], and they characterized renal impairment regardless of albuminuria. The main objective of this study was to determine the prevalence of kidney disease in patients with T2DM, forms of renal disease (renal impairment, albuminuria, renal impairment without albuminuria, nonalbuminuric renal impairment and diabetic nephropathy) and associated variables in a large sample of patients in primary care.

## Methods

*Study design and organization* The Continuous Quality Improvement Gedaps program gathered information from primary care centres on the clinical markers and health outcomes a sample of patients. A detailed description of the evaluation methodology has been previously described [15]. During the last evaluation in 2007, several meetings around Catalonia were conducted to encourage the involvement in the study regardless of participation in previous evaluations, and to present changes in the data entry using a webpage (http://www.redgdps.org/index.php?idregistro=259).

Health providers were instructed to obtain a random sample from the medical records of patients with T2DM with more than six months follow-up from diagnosis. A total sample of 5 patients multiplied by the number of basic care units (physician/nurse) with a minimum of 30 patients per centre was required. The exclusion criteria were as follows: type 1 diabetes mellitus; follow-up exclusively conducted by an endocrinologist; and short life expectancy (terminal patients or those who received home care). Only patients with plasma creatinine available were included for the current study.

Due to the retrospective nature of the study, based only on clinical records, patients were not required to give written informed consent. To assure anonymity data were collected and recorded using two different files: one included demographic variables and the other one clinical variables linked by a consecutive record number. The study design and the GCQI program were presented and approved by the Consell Assessor de la Diabetes (Advisory Board on Diabetes) of the Health Departament of the Autonomous Government in Catalunya that acted as Institutional Review Board.

**Figure 1:**
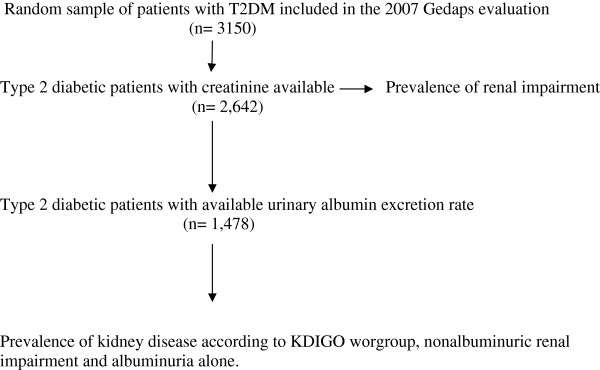
**Flow**-**chart of study.**

*Study population* The following demographic and clinical characteristics were compared: age, gender, weight, height, BMI, blood pressure, HbA1c, creatinine, total cholesterol, HDL cholesterol, albuminuria, duration of diabetes (in years), estimated cardiovascular risk according to the Framingham equation calibrated to the Spanish population [16] (threshold & 10% at 10 years), antidiabetic treatment, antihypertensive treatment, lipid-lowering therapy, antiplatelet therapy, and smoking status. Obesity was considered if the BMI was greater than 30 kg/m^2^. We found 1164 type 2 diabetic patients who had no available albuminuria. Figure 1 shows the samples used to calculate the prevalence of the different forms of kidney disease.

The following outcomes were considered: diabetic foot (ulcers and amputations), retinopathy, coronary heart disease (CHD, including angina), and stroke (including transient ischemic attack). Two composite variables including all microvascular complications (albuminuria and/or retinopathy) and macrovascular complications (CHD and/or stroke) were separately created. All collected data included the last value recorded in the medical records during 2007.

NKF [1] defines chronic kidney disease by the persistence of a low GFR and/or albuminuria during a period of at least three months. In our study, since no previous measurement of creatinine or albuminuria was available, it was impossible to know whether or not these conditions had persisted longer than three months. Thus, we defined kidney disease based and renal impairment based on a single determination. Serum creatinine was measured on a multiparameter analyzer (Cobas 711, Roche Modular System, Indianapolis, IN, USA) by the Jaffé Method with bichromatic measurements according to the manufacturer’s specifications. Albuminuria was measured by inmmunoturbidimetry on a Cobas 400 analyzer (Roche, Indianapolis, IN, USA).

The GFR was calculated using the equation previously published by the MDRD study group [17]. Variables of renal function were defined as follow: 1) Microalbuminuria: Albumin excretion rate (AER) & 30 mg/g (3.5 mg/nmol), 2) Macroalbuminuria: AER & 300 mg/g (35 mg/nmol), 3) Kidney disease: GFR < 60 ml/min/1.73 m2 and/or the presence of micro or macroalbuminuria, 4) Renal impairment: GFR < 60 ml/min/1.73 m2, 5) Nonalbuminuric renal impairment: GFR < 60 ml/min/1.73 m^2^ without micro or macroalbuminuria and, 5) Diabetic nephropathy (DN): macroalbuminuria or microalbuminuria plus diabetic retinopathy [18].

Patients with available creatinine and UAER were also classified according to the recent proposal of KDIGO workgroup [19].

*Statistical analysis* Quantitative variables were described using means and standard deviations. Categorical variables were summarised using absolute and relative frequencies (i.e., percentages). Bivariate relationships between categorical variables were tested using non-parametric chi-squared tests. Bivariate relationships between categorical and quantitative variables were assessed using ANOVA tests (one factor). Descriptive statistics and bivariate inferences were performed using the SPSS (v. 15) statistical program. The multivariate analyses of variables associated with different types of kidney disease in patients with T2DM were assessed by means of logistic regressions. The variables for the adjustment were selected on the basis of significant bivariate relationships. Response variables were assigned a value of 1 when the particular type of kidney disease (i.e., renal impairment, DN and kidney disease) occurred, and patients without any type of kidney disease were assigned a value of 0. The estimation of the logistic regressions was performed using the R freeware statistical package (version 2.11.1).

## Results

### Sample characteristics

From a total sample of 3150 patients with T2DM included in the 2007 Gedaps evaluation, we included 2,642 patients who have a plasma creatinine available. Mean age was 68 years (SD 11.6), and 51.5% of the patients were men. The average duration of diabetes was 7 years (SD 5.6). The mean HbA1c was 6.84% (SD 1.46), and 67.6% of patients had an HbA1c value less than or equal to 7%. The mean blood pressure was 137/76 mmHg. Table 1 shows the main characteristics of the population. To rule out a bias attributable to patients without albuminuria data, we compared a subgroups with available albuminuria data (1,478 patients), and to the 1,164 patients not having albuminuria data. Of all variables compared (age, gender, years of evolution, HbA1c, systolic blood pressure, diastolic blood pressure and creatinine), only age showed differences (67.59 vs 68.58; p = 0.03).

**Table 1:** **Characteristics of the sample of patients with type 2 diabetes** (**n** = **2**,**642**)

	**Total**	**Men**	**Women**	**p value**
Age, years; mean (SD)	68.06 (11.60)	66.81 (11.63)	69.31 (11.59)	< 0.001
Gender,n (%)	2642	1361 (51.5)	1281(48.5)	ns
Years of diabetes duration, mean (SD)	7.05 (5.60)	6.58 (5.06)	7.39 (6.04)	<0.001
Diabetes duration 5–10 years, n (%)	803 (30.4)	413 (30.4)	390 (30.5)	ns
Diabetes duration & 10 years, n (%)	497 (18.8)	231 (17.0)	266 (20.8)	< 0.05
BP, mmHg,mean (SD)	137.23/76.52 (14.60/9.05)	136.98/76.49 (14.68/9.10)	137.08/76.43 (14.71/8.89)	ns SBP ns DBP
Hypertension,n (%)	1847 (69.9)	894 (65.6)	953 (74.3)	<0.001
BP ≤ 140/90 mmHg,n (%)	1896 (71.7)	976 (71.7)	920 (71.8)	ns
BP ≤ 130/80 mmHg,n (%)	915 (34.6)	460 (33.8)	455 (35.5)	ns
HbA1c, mean (SD)	6.84 (1.46)	6.83 (1.47)	6.85 (1.44)	ns
HbA1c ≤ 7%, n (%)	1786 (67.6)	920 (67.6)	866 (67.6)	ns
HbA1c ≤ 8%,n (%)	2319 (87.7)	1182 (86.8)	1137 (88.7)	ns
Total cholesterol,mg/dl,mean (SD)	194.33 (38.41)	188.06 (38.45)	201.00 (37.25)	<0.001
Non-HDL cholesterol, mg/dl,mean (SD)	144,10 (37.44)	140.63 (37.72)	148.10 (36.65)	<0.001
Non-HDL cholesterol < 130 mg/dl, n (%)	1007 (38.1)	592 (43.5)	415 (32.4)	<0.001
Non-HDL cholesterol < 160 mg/dl, n (%) (1)	1802 (68.2)	957 (70.3)	845 (66.0)	0.025
Obesity, n (%)	1118 (42.3)	471 (34.6)	647 (50.5	<0.001
Tobacco habit, n (%)	365 (13.8)	299 (22.0)	66 (5.1)	<0.001
Creatinine, mg/dl (SD)	1.21 (1.03)	1.30 (1.12)	1.10 (0.92)	<0.001
GFR, mil/min/1.73 m^2^ (SD)	76.30 (32.79)	79.73 (29.48)	72.71 (35.67)	<0.001
Macrovascular disease,n (%) (1)	460 (17.4)	275 (20.2)	185 (14.4)	<0.001
CHD,n (%)	329 (12.4)	197 (14.5)	132 (10.3)	0.014
Stroke,n (%)	188 (7.1)	102 (7.5)	86 (6.7)	ns
Diabetic foot, n (%) (2)	100 (5.0)	48 (4.6)	52 (5.3)	ns
Microvascular disease (3)	475 (32.1)	260 (34.1)	215 (30.0)	ns
Albuminuria, n (%) (3)	288 (19.5)	184 (24.1)	104 (14.5)	<0.001
Diabetic retinopathy, n (%)	227(15.3)	113 (14.8)	114 (16.0)	ns
ACEI/ARB treatment, n (%)	1453 (54.9)	741 (54.5)	712 (55.5)	ns
Calibrated Framingham cardiovascular risk equation & 10%, n (%)	240 (9.1)	194 (14.2)	46 (3.6)	<0.001

(1) Coronary heart disease (CHD) and/or stroke; (2) Ulcers and/or amputations, n = 2,000, men 1030, women 970; (3) Albuminuria and/or diabetic retinopathy, n = 1478, men 761, women 717.

*BP*: blood pressure, *HDL*: high density lipoprotein, *GFR*: glomerular filtration rate, *ACEI*: angiotensin converting enzyme inhibitor, *ARB*: angiotensin receptor blocker.

### Prevalence of kidney disease

The prevalence of different types of renal disease was: 34.1% for kidney disease, 22.9% for renal impairment, 19.5% for albuminuria and 16.4% for diabetic nephropathy. Renal impairment according to the MDRD equation was found in 606 patients (22.9%) of whom 296 (11.2%) showed an elevated serum creatinine value (& 123.76 μmol/dl in women and & 132.6 μmol/dl in men). Moreover, 382 patients (14.5%) showed an estimated GFR between 30 and 59 ml/min/1.73 m2, and 224 patients (8.4%) of the total showed an estimated GFR less than 30 ml/min/1.73 m2. Additional file 1: Table S2 shows the prevalence of kidney disease as defined by the KDIGO workgroup [19] in 1478 T2DM patients in which the creatinine and albuminuria was available. Among patients with RI, 71.1% were nonalbuminuric.

There were significant differences in gender among the forms of kidney disease. Women had a significantly higher prevalence of kidney disease and renal impairment, and men had a higher prevalence of albuminuria and DN. Significant differences in the forms of kidney disease were also found with the duration of diabetes (Table 2).

**Table 2:** Prevalence of different types of kidney disease stratified by gender and years of Type 2 diabetes duration

	**Total**	**Men**	**Women**	**p value**	<**5 years diabetes duration**	≥**5 years diabetes duration**	**p value**
**KD**, **n** (%) (**1**)	505 (34.1)	235 (15.9)	270 (18.2)	0.028	327 (22.1)	178 (12.0)	<0.001
**RI**, **n** (%) (**2**)	606 (22.9)	239 (9.0)	367 (13.9)	<0.001	212 (8.0)	394 (14.9)	<0.001
**Diabetic nephropathy**, **n** (%) (**1**)	243 (16.4)	139 (9.4)	104 (7.0)	0.06	62 (4.2)	181 (12.2)	<0.001
**Albuminuria**, **n** (%) (**1**)	288 (19.5)	184 (12.4)	104 (7.1)	<0.001	97 (6.6)	191 (12.9)	<0.001

KD: kidney disease. Albuminuria and/or altered GFR; RI: renal impairment. GFR < 60 mil/min/1.73 m^2^. Diabetic nephropathy: Albuminuria & 300 mg/gr or albuminuria 30–300 mg/gr and retinopathy. Albuminuria: urinary albumin excretion rate & 30 mg/gr.

(1) n = 1478; (2) n = 2642.

**Figure 2:**
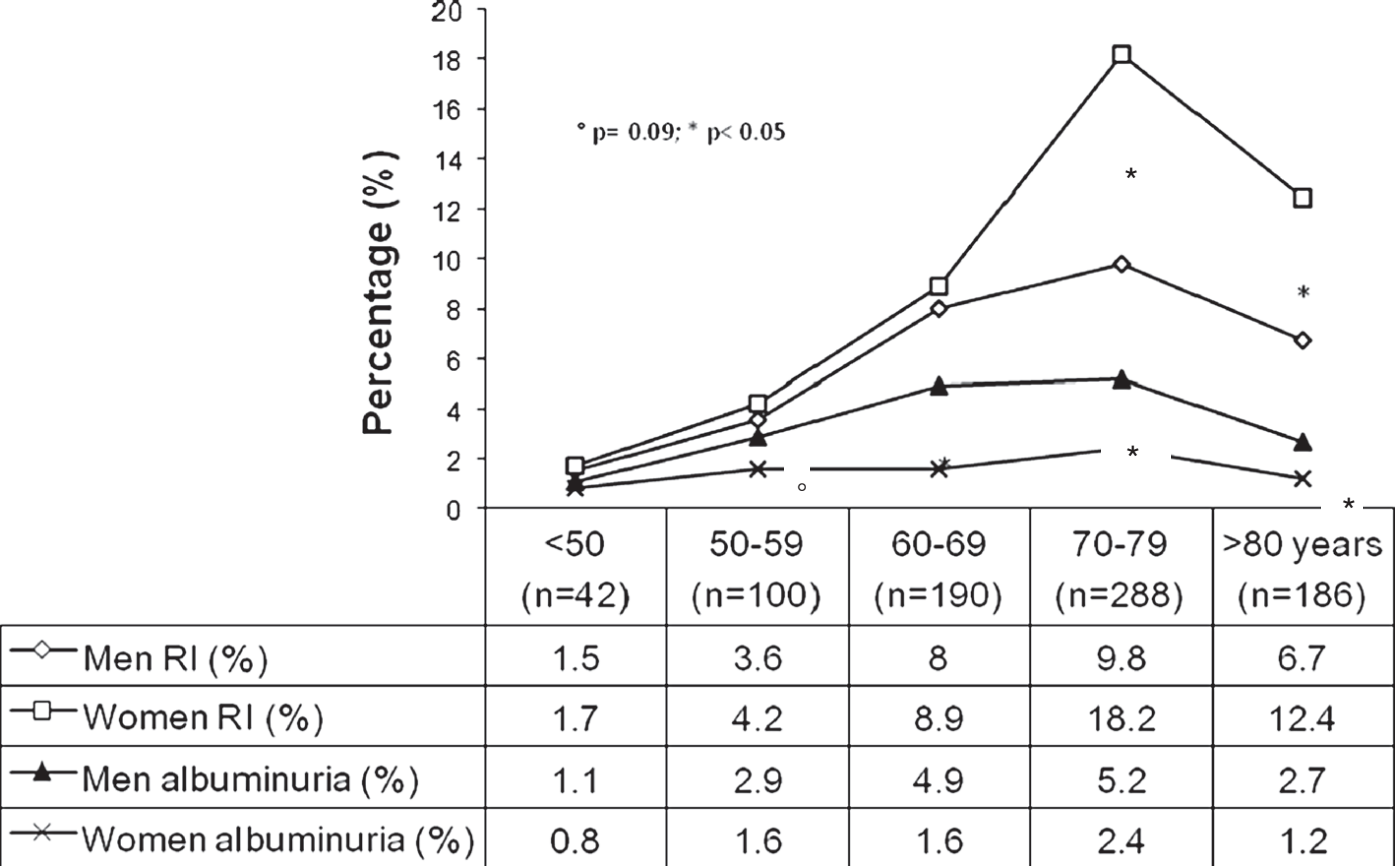
**Prevalence of renal impairment and albuminuria alone stratified according to age and gender(n** = **806)**.

Figure 2 shows that the prevalence of renal impairment was similar in both sexes until age 70 and that the prevalence of renal impairment was significantly higher in women after age 70. In contrast, the prevalence of albuminuria was higher in men in all age ranges.

### Types of kidney disease and associated variables

Among the 1,478 patients with T2DM who had available data for calculating both GFR and albuminuria, patients with albuminuria without renal impairment and nonalbuminuric renal impairment were compared. The prevalence of albuminuria without renal impairment (albuminuria alone) (13.5%) and nonalbuminuric renal impairment (14.7%) was similar. Relative to albuminuric patients without renal impairment, the patients with nonalbuminuric renal impairment were significantly older and with more probability to be women; they had a longer T2DM duration, lower prevalence of obesity, lower diastolic BP and lower HbA1c; and had a lower prevalence of smoking, macrovascular disease and ischemic heart disease (Table 3).

**Table 3:** Prevalence of different types of chronic kidney disease and associated variables in the 1478 diabetic patients in wich CRI and albuminuria data were available

	**Albuminuria without renal impairment**	**Nonalbuminuric renal impairment**
**n**,(%)	200 (13.5)	217 (14.7)
**Age**, **mean** (**SD**)	67.49 (11.89)	72.35 (9.94)^**a**^
**Gender**, **men** (%)	137 (68.5)	75 (34.6)^**a**^
**Type 2 DM duration**, **years** (**SD**)	7.13 (4.89)	8.41 (6.43)^**a**^
**Obesity**, **n** (%)	75 (44.4)	79 (39.3)^**a**^
**BP**, **mmHg** (**SD**)	139.79/77.79 (15.22/9.35)	138.12/75.38 (14.22/8.72)^**b**^
**HbA1c**, **mean** (**SD**)	7.27 (1.61)	6.68 (1.42)^**a**^
**Total cholesterol**, **mean** (**SD**)	187.74 (37.38)	193.17 (36.66)
**Non**-**HDL cholesterol**, **mg**/**dl**, **mean** (**SD**)	141.75 (34.89)	141.81 (35.75)
**Creatinine**, **mg**/**dl** (**SD**)	0.87 (0.17)	2.38 (1.60)^**a**^
**GFR**, **mil**/**min**/**1**.**73 m**^**2**^ (**SD**)	92.49 (58.24)	36.55 (19.28)^**a**^
**Tobacco**, **n** (%)	34 (18.1)	15 (7.0)^**b**^
**Macrovascular disease**, **n** (%)	46 (23.8)	33 (15.4)^**a**^
**CHD**,**n** (%)	37 (19.2)	21 (9.8)^**a**^
**Stroke**, **n** (%)	10 (5.2)	15 (6.9)
**Diabetic retinopathy**, **n** (%)	23 (20.2)	25 (18.9)
**Insulin treatment**, **n** (%)	43 (22.0)	43 (19.9)
**Calibrated Framingham CV risk eq**. &**10**%, **n** (%)	14 (10.4)	7 (6.3)

*BP*: blood pressure; *HDL*: high density lipoprotein, *GFR*: glomerular filtration rate, *CHD*: coronary heart disease, *CV*: cardiovascular *RI*: renal impairment.

Albuminuria without RI vs. Nonalbuminuric RI: ^**a**^ p <0.001; ^**b**^ p < 0.05.

Adjusted multivariate analysis shows that those T2DM patients with renal impairment have a different profile to those with albuminuria alone. The variables associated with the difference between both types of KD were: gender, years of diabetes duration and HbA1c (Table 4). Diabetic nephropathy patients only show gender differences and increased use of insulin.

**Table 4:** Multivariate analysis of types of kidney disease and associated variables in type 2 diabetic patients

	**Renal impairment OR (CI 95%)**	**Nonalbuminuric RI OR (CI 95%)**	**Diabetic nephropathy OR (CI 95%)**
**Gender(men)women**	**2.20 (1.86–2.54)**	**0.27 (0.21–0.35)**	**0**.**56(0.40**–**0.78)**
**Diabetes duration(≤10) & 10 years**	**1.43 (1.18–1.74)**	0.66 (0.48–1.08)	0.80 (0.54–1.19)
**D Diabetes duration *For year***	**1.03 (1.02–1.05)**	**0.94 (0.91–0.97)**	0.97 (0.94–1.1)
**ACEI**/**ARB treatment(No)Yes**	**1.51 (1.27–1.79)**	**1.42 (1.09–1.84)**	0.98 (0.70–1.37)
**BP (≤ 130/80 mmHg) & 130/80 mmHg**	0.86 (0.69–1.06)	2.16 (0.90–3.57)	1.18 (0.60–2.32)
**HbA1c for each 1% of increase**	**0**.**85(0.80**-**0.91)**	**1**.**19(1.09**-**1.30)**	1.21 (0.96-1.37)
**Insulin treatment(No)Yes**	**1**.**82(1.39**-**2.38)**	**1**.**42(1.08**-**1.84)**	**4**.**17(2.33**-**7.44)**
**Microvascular disease(No)Yes**	**2**.**14(1.80**-**2.54)**	-	-

**In bold**: **p** <**0**.**01**;

Adjusted also for age, body mass index, total cholesterol and non-HDL cholesterol, pulse pressure and macrovascular disease.

*RI*: renal impairment. Glomerular filtration rate < 60 mil/min/1.73 m^2^. Diabetic nephropathy: albuminuria & 300 mg/gr or albuminuria 30–300 mg/gr plus retinopathy. Albuminuria: albuminuria & 30 mg/gr.; *ACEI*: angiotensin-converting enyme inhibitor; *ARB*: angiotensin receptor blocker; *BP*: blood pressure; *HbA1c*; glycated haemoglobin; Microvascular disease: albuminuria and/or diabetic retinopathy.

## Discussion

Within a random sample of patients with T2DM living in Catalonia (Spain), the prevalence of kidney disease, renal impairment, albuminuria and diabetic nephropathy was found to be 34.1%, 22.9%, 19.5% and 16.4%, respectively. The prevalence of albuminuria without renal impairment and nonalbuminuric renal impairment was 13.5% and 14.7%, respectively. In the case of renal impairment, the estimation of GFR identified an additional 13.4% of patients with T2DM with renal impairment who would not have been diagnosed only with the measurement of plasma creatinine. The GFR estimation allowed the detection of 20.6% of patients with kidney disease in addition to the 13.5% of patients with albuminuria alone. When using strict criteria to define DN [18], the prevalence decreased from 19.5% (albuminuria) to 16.3% (albuminuria and diabetic retinopathy). Thus, the difference between these two approaches was only 3.1%. In our study, as recommended by the NKF-K/DOQI the same cutoff for normal AER has been used for both sexes. In contrast, the European Societies of Cardiology and Hypertension [20] recommend adopting different normal values according to gender (<31 mg/g or 3.5 mg/mmol for men and <22 mg/g or 2.5 mg/mmol for women). So, i is possible that if we had used the European criteria, the prevalence of albuminuria would have been similar to the prevalence obtained by [12] in a similar cohort: 20.6%.

Previous studies have shown that renal impairment prevalence is between 10.2 (sample of T2DM patients in China) [21] and 36% [22] . Most studies have found a prevalence between 15 and 30% [12-14,23-26] depending on ethnic and demographic characteristics of the samples such as age, gender and BMI [27]. The prevalence of renal impairment in the Mediterranean population has been shown to be between 16.6 [12] and 31.3% [13]. These differences may be due to younger patients and lower percentage of women in the first study, and higher percentage of women in the second study. The prevalence of albuminuria in our study (19.5%) was lower than in other studies, ranging from 31.7 [12] to 61% [28]. This difference may be attributed to a greater number of younger obese men. In our study, the majority (71.1%) of T2DM patients with renal impairment had nonalbuminuric renal impairment, which was slightly higher than to previously reported prevalence [29,30]. The profile of patients with nonalbuminuric renal impairment was different from those with albuminuria; there was a higher proportion of women in patients with nonalbuminuric renal impairment, and these patients were older, had a higher prevalence of obesity, had better metabolic control with less use of insulin, were less likely to smoke and had a lower prevalence of macrovascular disease. These two forms of kidney disease in patients with T2DM may correspond to different profiles [9]. Renal impairment reflects the filtration capacity of the kidney, and albuminuria is associated with kidney disease and generalized vascular inflammation. Renal impairment is associated with the duration of diabetes, and albuminuria has an inverse association where the process of glomerular involvement related to diabetes is slower than the appearance of microalbuminuria. However, both albuminuria and low GFR are independently associated with CV morbidity, CV mortality and total mortality [8].

The relationship between the mechanisms responsible for the development of albuminuria and renal impairment are not clear. In keeping with the most relevant studies published to date, our results show that nonalbuminuric RI is associated with female gender [9,21,25,28,29]. Nonalbuminuric RI was associated with the time elapsed from the diagnosis of diabetes, which was previously reported by Retnakavaran et al. [9]. However, Penno et al. [30] relate the duration of diabetes to both nonalbuminuric RI and albuminuria alone. Several authors showed that patients with nonalbuminuric RI have lower levels of HbA1c [9,24] and lower prevalence of obesity [28] than those with albuminuria alone, as seen in our study. According to our results, the profile of T2DM patient with nonalbuminuric RI is a woman with a diagnose of diabetes made more than 10 years ago, low percentage of obesity, and good metabolic control measured by HbA1c. On the other hand, the patient with albuminuria alone is preferably male, with high percentage of obesity, and poor metabolic control. Diabetes duration was not significant in the latter case. There is no obvious explanation for this phenomenon other than that these diseases cause gender-specific vascular damage. We found only one study [30] comparing nonalbuminuric RI with albuminuria alone in T2DM patients. Their results are in agreement with ours in that male gender and HbA1c are associated with the presence of albuminuria alone. However, our study showed that the duration of diabetes and age are variables that differ in the two diseases whereas in the study of Penno et al. [30], age and duration of diabetes, were associated with the two forms of kidney disease. Our results may be helpful to discriminate between these two kidney diseases by taking into account the variables associated with each one of them. Several studies have reported a higher prevalence of microvascular damage (retinopathy and kidney disease) in hypertensive women [31,32], but no data have been published showing a higher prevalence of microvascular damage in women with type 2 diabetes. A recent retrospective study showed that DN appeared significantly earlier among males than females [33]. This finding may explain the gender difference we found in our cross-sectional study based on a single observation. However, different phenotypic expressions depending on gender cannot be excluded.

A multivariate analysis showed a clear association between renal impairment and microvascular disease. In contrast, the bivariate analysis showed a clear association between albuminuria and macrovascular disease although the association disappeared in the multivariate analysis. It is possible that small vessels in women are more likely to develop damage than the small vessels in men. If this finding is confirmed in further studies, women with type 2 diabetes should be closely monitored. Another possibility is that the glomerular filtration rate in women estimated by the MDRD equation [34] underestimates the GFR. Thus, the prevalence of renal impairment would be falsely increased in women. Even if renal function in women, is underestimated by the MDRD equation, a recent study has reported the predictive value for coronary heart disease of the glomerular filtration rate calculated by the MDRD equation in a cohort of patients with high prevalence of diabetes when is added to the estimated risk according to Framingham [35]. In this study, the predictive effect was independent of albuminuria in women, but albuminuria was the variable with predictive value in men.

This study had several limitations. First, in terms of internal validity, it should be noted that there was a single measurement of GFR. Therefore, it was not possible to distinguish between patients with transient impairment of GFR and those with a persistent alteration. To distinguish between these patients, a reassessment of the GFR at 3 months would be necessary. A previous study has shown that kidney disease detected by a single determination is also associated with increased CV risk although the association contributes to a lesser degree than the two measurements [36]. Second, although the sampling was random and well reflected the patients with type 2 diabetes treated in primary care in Catalonia (Spain), the determination of plasma creatinine was not performed by a single laboratory. All reference laboratories have used Jaffé alkaline picrate method as a technique study, the variation of which is 1.31% [37]. For this reason, we believe that it is highly unlikely that these small differences in the estimation of creatinine may have influenced the results. Finally, due to the study design, patients not visited in primary care in the past year were excluded from the study as well as patients who had no creatinine measurement available during the study period. These patients may have had less control of their diabetes, blood pressure and dyslipidaemia, which may have been caused by a lower adherence to the prescribed treatment. Therefore, these patients may have introduced some selection bias into the study.

## Conclusions

In summary, our results show that approximately one-third of type 2 diabetic patients in the Mediterranean area have kidney disease and that one-fourth of the patients have renal impairment, but only 10% would have been classified having renal impairment if the estimated glomerular filtration rate was not used. Moreover, one-fifth of the patients had albuminuria. T2DM patients with kidney disease have a different profile depending on the presence or absence of albuminuria or RI. Further studies are needed to identify the etiopathogenesis of these differences and may impact on the prevention and treatment of forms of kidney disease. Thus, it is important to determine AER and to estimate GFR to diagnose kidney disease and to initiate the appropriate treatment.

## Abbreviations

AER: Albumin excretion rate; CV: Cardiovascular; DN: Diabetic Nephropathy; GFR: Glomerular filtration rate; MDRD: Modification of Diet in Renal Disease; RI: Renal impairment; T2DM: Type 2 Diabetes Mellitus.

## Competing interest

The authors declare that there is no conflict of interest associated with the manuscript.

## Authors' contributions

G Coll de Tuero, M Mata-Cases, A Rodriguez-Poncelas and M Saez have participated in the conception and design, interpretation of data, drafting, revising and final approval of the work. JM Pepió, P Roura, B Benito and J Franch have participated in the conception and design, revising and final approval of the work. All authors read and approved the final manuscript.

## Pre-publication history

The pre-publication history for this paper can be accessed here:

http://www.biomedcentral.com/1471-2369/13/87/prepub

## Supplementary Material

Additional file 1 **Table S2.** Classification of patients with creatinine and albumin excretion rateavailable according to KDIGO 2009. Click here for file

Additional file 2 List of participating investigators in the GEDAPS 2007 Evaluation. Click here for file
