# Supplementary material for: Chronic kidney disease in the type 2 diabetic patients: prevalence and associated variables in a random sample of 2642 patients of a Mediterranean area
Source: BMC Nephrol. 2012 Aug 20;13:87. doi: 10.1186/1471-2369-13-87 (PMC3537582; doi:10.1186/1471-2369-13-87)
Supplement: Additional file 1 — Table S2. Classification of patients with creatinine and albumin excretion rateavailable according to KDIGO 2009. [file 1471-2369-13-87-S1.doc]

**Additional file 2. Classification of patients with creatinine and albumin excretion rate available according to KDIGO 2009**

|  | n (%) | | | Albuminuria stages (mg/g) | | | | |
| --- | --- | --- | --- | --- | --- | --- | --- | --- |
| GFR stages  (mil/min  per  1.73m^2^) |  |  |  |  | | | | |
|  |  |  |  | A1 | | A2 | A3 | All |
|  |  |  |  | Optimal and high normal | | High | Very high |  |
|  |  |  |  | < 10 | 10-29 | 30-299 | ≥ 300 |  |
|  | G1 | Increased and optimal | ≥ 105 | 128 (8.6) | 47 (3.2) | 31 (2.1) | 1 (0.06) | 207 (14.0) |
|  |  |  | 90-104 | 142 (9.6) | 42 (2.8) | 40 (2.8) | 4 (0.3) | 228 (15.4) |
|  | G2 | Mild | 75-89 | 269 (18.2) | 109 (7.4) | 71 (4.8) | 5 (0.3) | 454 (30.7) |
|  |  |  | 60-74 | 165 (11.1) | 71 (4.8) | 42 (2.8) | 6 (0.4) | 284 (19.2) |
|  | G3a | Mild-  moderate | 45-59 | 73 (5.0) | 36 (2.4) | 34 (2.3) | 9 (0.6) | 152 (10.3) |
|  | G3b | Moderate-severe | 30-44 | 20 (1.3) | 11 (0.7) | 12 (0.8) | 4 (0.3) | 47 (3.2) |
|  | G4 | Severe | 15-29 | 13 (0.9) | 20 (1.4) | 8 (0.5) | 2 (0.1) | 43 (2.9) |
|  | G5 | Kidney failure | <15 | 24 (1.6) | 20 (1.4) | 11 (0.7) | 8 (0.5) | 63 (4.3) |
|  | All | | | 834  (56.4) | 356  (24.1) | 249  (16.8) | 39  (2.6) | 1478 (100) |

Albuminuria alone

Nonalbuminuric RI

GFR: glomerular filtration rate
