# Supplementary material for: Chronic kidney disease in the type 2 diabetic patients: prevalence and associated variables in a random sample of 2642 patients of a Mediterranean area
Source: BMC Nephrol. 2012 Aug 20;13:87. doi: 10.1186/1471-2369-13-87 (PMC3537582; doi:10.1186/1471-2369-13-87)
Supplement: Additional file 2 — List of participating investigators in the GEDAPS 2007 Evaluation. [file 1471-2369-13-87-S2.pdf]

Appendix List of participating investigators in the GEDAPS 2007 Evaluation

Mª Josepa Arasa Fava, Joan Francesc Barrot de la Puente, Belén Benito Badorrey, Mª Dolors Berenguer Iglesias, Martí Birulés Pons, Isabel Bobé Molina, Dolors Boix Pujol, Montserrat Boneu Castells, Mª Remei Bosch Costabella, Montserrat Brugada Mir, Magda Bundó Vidiella, Ricard Carrillo Muñoz, Carmen Ciria Mallada, María de Ciurana Gay, Francesc Xavier Cos Claramunt, Pilar Cosculluela Torres, Engracia Costa Atienza, Pilar Enseñat Grau, Assumpta Farràs Mas, Eugeni Fau Montllor, Pilar Font Roura, Pilar Franco Comet, Isabel García Villena, Josep Mª Garrido Martín, Jorge Gentille Lorente, Rosa Maria Gimbert Ràfols, Joan Carles González Pastor, Clementina Gonzalez Rosua, Neus Gregori Ortiz, Mª Cruz Guzmán Bonilla, Juan Herreros Melenchón, Cristina Laserna Jiménez, Carmen Lecumberri Pomar, Montserrat Ledesma Joanmiquel, Judit Llussà Arboix, Regina López López, Mª Victoria Marina Ortega, José Antonio Martín García, Ana I. Martínez Asensio, Daniel Martínez Laguna, Pilar Martínez Marco, Manel Mata Cases, Ana Menal García, Mª Angeles Méndez Trías, José Mercader Coma, F. Xavier Mestres Gallinat, Rosa Mar de Miguel Pérez, Xavier Mundet Tuduri, Marifé Muñoz Grimaldo, Rosa Blanca Muñoz Muñoz, Isabel Otzet Gramunt, Pilar Pallarés Perna, María Pastoret Descamps, Ramón Pujol Martínez, Mónica Rodríguez Barniol, José Osvel Rodríguez Barrios, Antonio Rodríguez Poncelas, Olivia Roig Calvet, Pilar Roura Olmeda, Laura Rubio Pérez, Montserrat Rubio Villar, Rou Sánchez Collado, M. Carmen Soldado Ordóñez, Àngels Sieira Ribot, Ana Mª Suárez Sanjuas, Marta Serra Laguarta, Marta Torné Coll, Anna Torné Coromina, Cristina Verdera Benedicto, Elena Villanueva Santamaría
